# The Role of Fatty Acid Oxidation in the Metabolic Reprograming of Activated T-Cells

**DOI:** 10.3389/fimmu.2014.00641

**Published:** 2014-12-18

**Authors:** Craig Alan Byersdorfer

**Affiliations:** ^1^Department of Pediatrics, Division of Blood and Marrow Transplantation and Cellular Therapies, University of Pittsburgh, Pittsburgh, PA, USA

**Keywords:** T-cell metabolism, fatty acid oxidation, oxidative phosphorylation, reactive oxygen species (ROS), AMP-activated protein kinase, graft-versus-host disease, *in vivo* models

## Abstract

Activation represents a significant bioenergetic challenge for T-cells, which must undergo metabolic reprogramming to keep pace with increased energetic demands. This review focuses on the role of fatty acid metabolism, both *in vitro* and *in vivo*, following T-cell activation. Based upon previous studies in the literature, as well as accumulating evidence in allogeneic cells, I propose a multi-step model of *in vivo* metabolic reprogramming. In this model, a primary determinant of metabolic phenotype is the ubiquity and duration of antigen exposure. The implications of this model, as well as the future challenges and opportunities in studying T-cell metabolism, will be discussed.

## Introduction

Activation precipitates a dramatic change in T-cell physiology. Upon stimulation, T-cells increase their DNA replication, synthesize cytokines, and upregulate multiple signaling pathways (1, 2). Proliferation increases exponentially, with stimulated cells dividing as frequently as every 4–6 h at the height of an immune response (3, 4). The energetic requirements for these new tasks dictate that T-cells must undergo metabolic reprograming in order to generate sufficient biomass and produce adequate adenosine triphosphate to meet the increased metabolic demands (5).

In recent years, increasing attention has focused on the metabolic pathways adopted by T-cells following activation. Many fine contemporary reviews highlight the relationship between metabolic phenotype and signal transduction (6, 7), T-cell differentiation (8, 9), and T-cell function (5, 10). Other reviews stand as thorough summaries on overall T-cell metabolism, and the reader is encouraged to seek out these important works (11). This review will focus on the use of fatty acid oxidation (FAO) by activated T-cells, both *in vitro* and *in vivo*, and suggest a possible connection between the environment present during activation and adoption of this alternative metabolic pathway. To place the findings on FAO into a contextual framework, I will begin by briefly reviewing the role of other metabolites, including glucose and amino acids, in T-cell metabolism.

## The Necessity of Glycolysis and Amino Acids

Early studies demonstrated increased rates of both glycolysis and lactate production during mitogen activation of rat thymocytes, suggesting a prominent role for glucose metabolism during *in vitro* T-cell stimulation (12). Following activation, T-cells increase multiple steps in glucose metabolism, including upregulation of the glucose transporter Glut1, in a highly regulated process that is at least partially dependent upon signaling through the co-stimulatory molecule CD28 (13–15). Failure of T-cells to sufficiently increase glucose metabolism decreases both proliferation and cytokine production, while overexpression of a transgenic Glut1 receptor increases cytokine production and improves T-cell survival (16, 17).

Glutamine metabolism is also requisite during T-cell activation and limiting glutamine in the culture media decreases proliferation and cytokine production in mitogen-stimulated lymphocytes (18). Studies on purified populations of T-cells confirmed the importance of glutamine uptake during *in vitro* stimulation and implicated a role for CD28 in maximizing glutamine uptake (19). In addition, inflammatory CD4 T-cell responses depend on glutamine uptake through expression of the amino acid transporter Slc1a5 (20), and absence of Slc1a5 decreases the percentage of IFN-γ^+^ T-cells responding to *Listeria monocytogenes* infection. Similarly, the transcription factor Myc plays a pivotal role in directing glutamine into obligate biosynthetic pathways and facilitates the initial proliferative burst (21). Thus, both glucose and glutamine appear indispensable for early events in T-cell metabolic reprograming.

In addition to glutamine, T-cells require access to other amino acids for proliferation and survival. Expression of the bidirectional glutamine/leucine transporter Slc7a5 is an integral event in early T-cell activation and absence of this receptor decreases T-cell responses both *in vivo* and *in vitro* (22). The importance of this receptor is intriguing, given that leucine is a necessary component of T-cell activation and that higher glutamine levels facilitate leucine import through simultaneous glutamine export (23). Therefore, a large role for glutamine may simply be to provide an intracellular gradient to support transport of other amino acids. This hypothesis is supported by the finding that glutamine transporter deficiency can be overcome through increasing concentrations of leucine. Additional data suggest that glutamine transport may even initiate metabolic adaptation, as absence of Slc1a5 in T-cells blunts expression of other metabolic mediators including both Glut1 and CD71 (22).

In addition to leucine, T-cells also depend upon tryptophan to execute full effector function. Suppressed T-cell responses are observed when antigen presenting cells contain indolamine 2,3-dioxygenase (IDO), an enzyme that catabolizes tryptophan (24, 25). The importance of the IDO pathway has been demonstrated in multiple immunogenic processes, including fetal tolerance during pregnancy, bone marrow transplantation, antitumor responses, and autoimmunity (26). In addition, kynurenine, a tryptophan catabolite, induces regulatory T-cell generation (T_reg_) through its action on the aryl-hydrocarbon receptor (21). Thus, T-cell responses can be modulated by both decreased levels of a nutrient (tryptophan) and the actions of its metabolic derivative (kynurenine).

## Nutrient Regulation of T-Cell Differentiation

Other nutrients also influence T-cell differentiation and function. Short-chain fatty acids, such as propionate and butyrate, are generated via fermentation by intestinal bacteria and intestinal levels of these fatty acids also modulate T_reg_ formation (19, 27, 28). Similar to kynurenine, propionate and butyrate likely drive T_reg_ formation through specific intestinal T-cell nutrient receptors, but the precise mechanism has yet to be confirmed. In a similar way, Vα9^+^Vδ2^+^ gamma-delta T-cells selectively respond to the microbial metabolite (E)-4-hydorxy-3-methyl-but-2-enyl pyrophosphate (29). High salt concentrations also affect T-cell function and drive CD4 T-cells toward a Th17 phenotype both *in vivo* and *in vitro*. This is clinically relevant because dietary increases in salt worsen the severity of experimental autoimmune encephalomyelitis in murine models (30, 31). Salt sensitivity in Th17 cells occurs via increased expression of serum glucocorticoid receptor-1, a protein, which governs sodium homeostasis in multiple cell types (32). Thus, extracellular nutrients not only help meet increased energy needs of activated T-cells, but may also dictate their differentiation and effector status.

## The Role of Fatty Acid Oxidation

Oxidation of fat, in addition to the catabolism of glucose and glutamine, was first implicated as an energy source in unstimulated lymphocytes (33), although most studies suggest that naïve T-cells require only a minimal rate of metabolism to meet their bioenergetics needs (5, 34). A role for FAO in other subsets first came from work in T-cells bearing a deletion of TNF receptor associated factor 6 (TRAF6). CD8 T-cells deficient in TRAF6 are unable to form memory cells in response to infection with *L. monocytogenes* and when taken *ex vivo*, decrease rates of β-oxidation (35). Furthermore, indirect activation of AMP-activated protein kinase (AMPK), a cellular energy sensor and controller of FAO (36–38), increased CD8 memory T-cell generation and improved survival in a lethal tumor model. Subsequent *in vitro* work demonstrated that IL-15, an important cytokine in memory T-cell generation, upregulates expression of carnitinepalmitoyltransferase 1a (CPT1a), the rate limiting enzyme in FAO (39). These studies suggest a subset specific role for FAO in the generation of CD8 memory T-cells.

CD4 T-cells cultured *in vitro* also exhibit a subset specific dependence on FAO. T-cells differentiated *in vitro* toward Th1, Th2, or Th17 profiles adopt a glycolytic phenotype, consistent with earlier findings on T-cell metabolism (13, 14). In contrast, T_regs_ generated *in vitro* increase lipid oxidation and phosphorylate AMPK. Furthermore, *in vitro* blockade of FAO with the CPT1a inhibitor etomoxir disrupts T_reg_ generation and *in vitro* supplementation with fatty acids supports T_reg_ function (17). *In vivo* administration of metformin increases both the percentage and total number of T_reg_ during a murine model of asthma (17).

Together, these data support a mandatory role for FAO in both IL-15 driven CD8 T-cell responses and in the induction of *in vitro* generated T_regs_. Furthermore, metformin administration, which indirectly activates AMPK, increases both T_reg_ and memory CD8 T-cells, and could indicate a role for AMPK in controlling FAO in these cell types (17, 35). This notion is supported by the fact that CD8 T-cells deficient in AMPKα1 mount inferior memory T-cell responses following *L. monocytogenes* infection (40). It remains unclear, however, exactly how metformin increases T_reg_ and CD8 memory T-cells and the extent to which AMPK controls pathways of T-cell metabolism beyond FAO (41–43).

Metformin is a direct inhibitor of Complex I of the electron transport chain (44) and through inhibition of oxidative phosphorylation can indirectly accelerate glycolysis (45). However, it is unlikely that increased glycolysis drives memory T-cell formation, as glycolytic inhibition has already been shown to increase CD8 memory T-cell generation (46). However, the rapid recall response of memory T-cells requires an imprinted glycolytic potential (47), suggesting that the transition from memory to effector phenotype depends upon glycolysis and is, therefore, potentially influenced by the indirect effects downstream of metformin. Further clarity on the direct role of AMPK in driving FAO and will be gained using more selective inhibitors of AMPK and genetically deficient animal models (43, 48).

One of the key challenges in studying immune cell metabolism *in vitro* is the relevance of experimental systems to the environmental conditions encountered *in vivo* (10, 49). Standard culture concentrations differ greatly from physiologic values, including higher concentrations of glucose (three- to fivefold higher than the standard serum glucose of 5 mM), glutamine (eightfold higher than serum levels), and oxygen (21% in culture compared to 2–5% *in vivo*) (50). Changes in these environmental variables can affect both a cell’s function and metabolic response (51). For example, decreased glucose availability modulates both oxygen consumption and metabolic transcription factors during human CD4 T-cell activation (52). Hypoxia reduces proliferation and cytokine production and promotes glycolysis (53, 54). Thus, the metabolic phenotype adopted by a T-cell following *in vitro* stimulation may be very different from the phenotype adopted by T-cells activated under physiologic conditions *in vivo* (55).

One attractive approach to this challenge is to study metabolic reprograming in lymphocytes activated *in vivo* during graft-versus-host disease (GVHD). During GVHD, alloreactive donor T-cells respond robustly to the presence of host antigens, leading to marked proliferation, destruction of host tissues, and profound inflammation (56–58). Allogeneic T-cells taken directly from GVHD animals demonstrate a 2.5-fold increase in oxygen consumption (a surrogate for oxidative phosphorylation) and a modest increase in the expression of Glut1 (59). Increased oxidative metabolism during GVHD is consistent with data from patients with systemic lupus erythematosus, where isolated T-cells increase their mitochondrial mass by 50% and expand their mitochondrial membrane potential by 20%. Similarly, peripheral blood mononuclear cells from these patients increase oxygen consumption by 50% compared to healthy control cells (60, 61).

Recent studies suggest a direct role for FAO in effector T-cells during GVHD. Levels of acylcarnitines, necessary intermediates in the oxidation of fat (62), increase 10-fold or more in allogeneic T-cells by day 7 post-transplant (59). In addition, effector GVHD T-cells (characterized by their CD44^hi^, CD62^Lo^ phenotype) increase fatty acid transport, upregulate levels of CPT1a and CPT2, and increase their rates of FAO *ex vivo* (63). Treatment of allogeneic cells with etomoxir selectively decreases their proliferation *in vitro*, while etomoxir administration *in vivo* decreases both total donor T-cell numbers and the severity of GVHD (63). In contrast, T-cells proliferating in a homeostatic fashion after transplantation, and those responding to cellular immunization, minimally increase fatty acid transport and display no sensitivity to etomoxir (63). Thus, FAO appears to be a specific metabolic adaptation in effector T-cells proliferating in response to large quantities of antigen. This is supported by findings in an experimental autoimmune encephalomyelitis model, where etomoxir blockade of FAO decreased disease severity, limited demyelination, and reduced effector cytokine production (64).

Consistent with an increase in oxidative phosphorylation, allogeneic T-cells also increase generation of reactive oxygen species (ROS) (59). Increased ROS likely result from increased mitochondrial membrane potential (Δψ*m*), which prolongs the half-life of reactive intermediates in the electron transport chain, leading to increased leak of single electrons from the intra-mitochondrial space and subsequent formation of ROS (65). The increased ROS observed in allogeneic T-cells is also consistent with data from patients with systemic lupus erythematosus, where T-cells exhibit both hyperpolarization of the Δψ*m* and increased ROS (61). In addition, increases in ROS and oxidative phosphorylation can be therapeutically targeted, as modulators of complex V of the electron transport chain mitigate the severity of GVHD without affecting homeostatic reconstitution (59).

**Figure 1:**
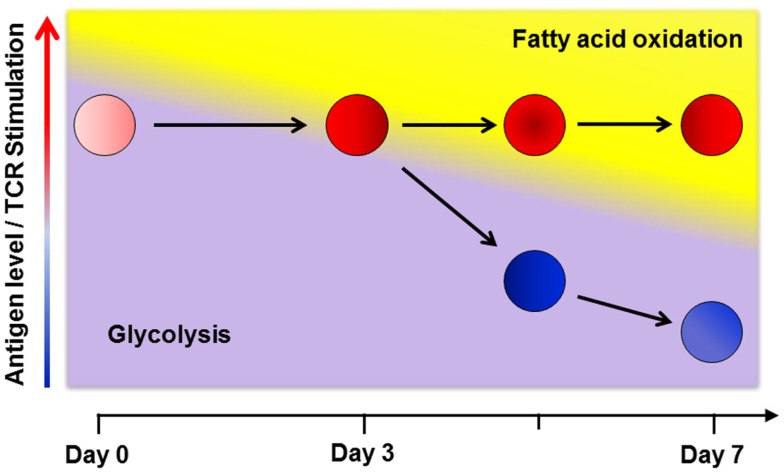
**A multi-step model of *in vivo* metabolic reprograming**. Early in a graft-versus-host (GVH) response, T-cells use glycolysis, glutaminolysis and glucose oxidation to meet their short term energy needs (16, 21, 67). By day 3 post-transplant, robustly activated cells (shown in red) require additional metabolic reprograming to keep pace with ongoing energetic demands and so upregulate fatty acid oxidation (FAO) by increasing fat uptake, turning on co-activator molecules, and upregulating fatty acid oxidation enzymes. This transition comes with a concomitant rise in reactive oxygen species and a moderation in the rate of glycolysis (63, 68). In contrast, T-cells stimulated via cellular immunization, with a limited duration of antigen exposure, only transiently increase fat uptake and ROS on day 3. As antigen levels fall, stimulation decreases and cells no longer require FAO (cells shown in blue). Levels of oxidation enzymes, co-activator molecules, fat transport, and ROS levels decrease to baseline in these cells (63). Thus, despite similar CD44^Hi^CD62L^Lo^ effector profiles, the metabolic phenotype on day 7 is clearly different between robustly and transiently activated T-cells. From these data, I propose that a primary determinant of metabolic reprograming in effector T-cells is both the degree and duration (< or >3 days) of antigen exposure at the time of evaluation.

The increased glycolysis in T-cells during GVHD is modest compared to the level of glycolysis and glucose uptake observed during *in vitro* activation (14, 55). Calculations based upon O_2_ consumption suggest a larger contribution from oxidative metabolism toward total energy production in allogeneic T-cells (66). This disparity might be accounted for by differences between *in vitro* and *in vivo* conditions, as described earlier (52). However, this explanation fails to account for situations where *in vivo* effector T-cells do not upregulate fatty acid transport following cellular immunization or increase mitochondrial mass following infection, as might be expected from cells with increased oxidative metabolism (39, 63). To unify the disparate results both between *in vitro* and *in vivo* conditions, and across different *in vivo* scenarios, I propose a multi-step model of metabolic reprograming in T-cells (Figure 1), where a primary determinant of metabolic phenotype is both the duration and degree of environmental stimulation present at the time of analysis.

In setting up this model, it is important to note that T-cells during GVHD do not increase fatty acid transport until their fifth division, which occurs on approximately day 3 post-transplant (63). Thus, early events in T-cell activation, even in the presence of significant antigen, do not require additional fatty acids. Instead, early during a graft-versus-host (GVH) response, T-cells likely utilize glycolysis and glutaminolysis to meet their short-term energy needs (16, 21). This idea is supported by data obtained following *in vivo* administration of the superantigen staphylococcal enterotoxin B (SEB), where SEB sensitive CD4^+^, Vβ8^+^ cells undergo a 15-fold increase in glycolysis 48 h post-administration of SEB (21). Initial dependence on glycolysis also explains the early *in vivo* sensitivity of T-cells to the glycolysis inhibitor 2-deoxyglucose (67).

**Figure 2:**
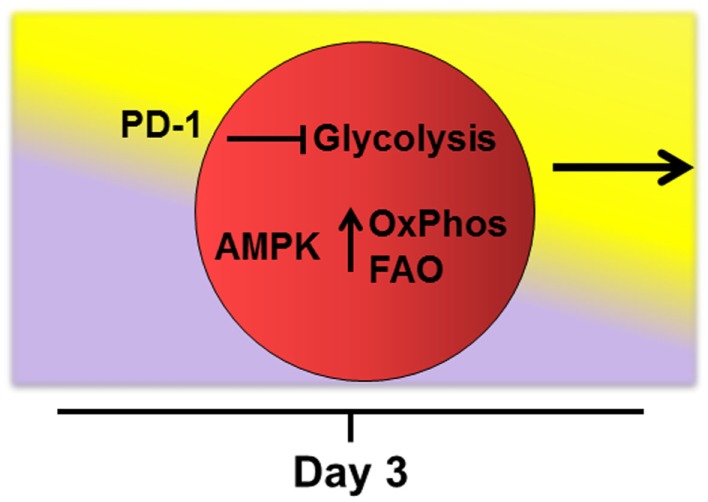
**Potential drivers of fatty acid oxidation**. Mechanisms that drive transition of robustly activated T-cells toward oxidative metabolism and fatty acid oxidation remain undefined. The checkpoint molecule PD-1 is upregulated early on allogeneic T-cells and PD-1 expression tracks with levels of reactive oxygen species during GVHD (manuscript in preparation). In addition, signaling through PD-1 is known to restrict T-cell glycolysis (70). AMPK acts as a “cellular energy sensor” and may respond to increasing AMP/ATP ratios as cells proliferate beyond 4–5 cell divisions. AMPK promotes oxidative metabolism (OxPhos) and FAO in multiple systems (36, 37, 71, 72) and knockout of AMPKα1 increases glycolytic metabolism in T-cells (73). Thus, activated AMPK might simultaneously dampen T-cell glycolysis while promoting adoption of FAO during periods of persistent antigen activation.

Later in the response (i.e., after 4–5 cell divisions), robustly activated T-cells require additional reprograming to keep pace with the ongoing demands of persistent activation (68). This reprograming includes increased fatty acid uptake, upregulation of oxidation enzymes and co-activator molecules, moderation in the rate of glycolysis, and adoption of FAO with a concomitant rise in ROS. This view is consistent with effector T-cells maintaining oxidative phosphorylation following activation under a variety of activating conditions (21, 39, 69). Mechanisms that drive this second metabolic transition remain undefined, but signaling through PD-1 is known to restrict glycolysis (70). In addition, AMPK promotes FAO in multiple systems and is known for its ability to act as a “cellular energy sensor” (36, 37, 71, 72). Indeed, knockout of AMPKα1 increases Glut1 expression, hexokinase levels, and glycolytic metabolism in purified T-cells. These observations suggest that when present, AMPK might actively dampen T-cell glycolysis (73), perhaps at the cost of promoting FAO (Figure 2).

Implicit in the multi-step model is the idea that continued stimulation of the T-cell receptor (TCR) drives later stages of metabolic reprograming. As antigen levels fall during the resolution of an immune response, stimulation decreases, energetic demands shrink, and effector T-cells no longer require utilization of alternative energy sources. Transgenic OT-I T-cells, when transferred into irradiated recipients that bear ovalbumin as a self-protein, markedly increase fat transport, ROS levels, and markers of oxidative metabolism. In contrast, when the same OT-I T-cells are stimulated by immunization with OVA-bearing dendritic cells, they return to baseline values of fat transport and oxidative metabolism by day 6 post-immunization (63). In these models, differences between GVHD and immunization responses were not dependent on differentiation status of the responding OT-I cells. Cells from either environment had similar CD44^Hi^CD62^Lo^ profiles and made equivalent amounts of IFN-gamma upon re-stimulation, consistent with the ability to separate effector function and metabolic phenotype on a per cell basis (74).

Although allogeneic T-cells only modestly increase glycolysis (59), glucose is still likely being utilized by these cells in alternative pathways. Indeed, shunting of glucose through the pentose phosphate pathway increases levels of reducing equivalents through production of NADPH and also generates building blocks for nucleic acid synthesis (75). In addition, glucose derivatives can be combined with oxaloacetate in the TCA cycle to form citrate, exported back to the cytosol via the carnitine/palmitate shuttle, converted into acetyl-CoA and then malonyl-CoA via the action of acetyl-CoA carboxylase 1 (ACC1), and eventually incorporated into *de novo* lipid synthesis. Evidence that a similar process occurs in activated T-cells comes from work using ACC1 deficient T-cells. CD8 T-cells lacking ACC1 have impaired survival following *L. monocytogenes* infection (74) and ACC1 deficiency preferentially disrupts *in vitro* differentiation of Th17 T-cells while simultaneously sparing development of T_reg_ (76). This Th17/T_reg_ dichotomy is reminiscent of studies on hypoxia-inducible factor 1-α and aryl-hydrocarbon receptor signaling, where promotion of Th17 responses occurs at the expense of T_reg_ generation (77, 78). In addition, both treatment with an ACC1 specific inhibitor and use of ACC1 deficient T-cells leads to diminished severity of EAE (76). However, in contrast to FAO inhibition, which preferentially affects antigen-activated T-cells (63), deficiency of ACC1 impacts both antigen-activated and homeostatic responses (74). This suggests that lipid synthesis, as driven by ACC1, is likely a necessity for proliferation of all T-cells.

Similar to the diversity of glucose metabolism, glutamine may, in addition to glutaminolysis, play a role as a metabolic substrate in one-carbon serine metabolism, as has been shown for proliferating cancer cells (79). Thus, as effector T-cells proliferate beyond 4–5 cell divisions with ongoing TCR stimulation, the roles of glucose and glutamine likely change, as increased fat transport feeds fatty acids into the TCA cycle. However, even fat-derived intermediates may exit the TCA cycle into alternative metabolic shunts (e.g., via the aspartate-malate shuttle) and the role of these divergent pathways during the latter stages of ongoing T-cell activation remain exciting areas for future investigation.

Given the ability of effector T-cells to reprogram their metabolic phenotype, the question arises as to whether T-cells can be pre-programed for the physiologic conditions they will encounter *in vivo*, a potential advantage when providing anti-viral or antitumor immunity. Recent work lends credence to this possibility. Treatment of *ex vivo* CD8 T-cells with the glycolysis inhibitor 2-deoxyglucose (2DG) increases phosphorylation of AMPK, heightens oxygen consumption, and decreases multiple markers of glycolysis in these cells. In addition, *ex vivo* treatment of cells with 2DG enhances their *in vivo* antitumor function (46). These data suggest that glycolytic inhibition *ex vivo* drives upregulation of alternative metabolic phenotypes, which then provide a subsequent selective survival advantage *in vivo*.

In contrast to allogeneic activation, T-cells responding via homeostatic proliferation minimally upregulate fatty acid transport and are not susceptible to FAO inhibition. This observation suggests that the metabolic demands of homeostatic renewal are distinct from those of T-cell activation, as has been seen in other studies of T-cell metabolism (22). These results also imply that modulating T-cell metabolism may offer a selective intervention against pathogenic cells, potentially leading to a decrease in overall immunosuppression (55, 59). From this perspective, it becomes critically important to understand at which stage of metabolic reprograming the intervention occurs. Some therapies, such as antithymocyte globulin, will eliminate all T-cells regardless of proliferation or activation status. Other treatments, like inhibition of ACC1, will affect survival of all proliferating T-cells, regardless of the stimulus (antigen-activated versus homeostatic cues) (74). Interventions that disrupt early events in TCR-activated metabolic reprograming (e.g., disruption of amino acid transport) might spare homeostatic T-cells, but will target T-cells whose metabolic reprograming is driven through TCR stimulation (80). Finally, interventions that affect the latter stages of metabolic adaptation, such as inhibition of FAO or modulation of oxidative phosphorylation, will likely only inhibit effector T-cells responding to prolonged antigenic stimulus (63). This last form of intervention may be particularly relevant in T-cells undergoing continuous exposure to antigen (e.g., during autoimmunity and following transplantation of bone marrow or solid organs) and highlights situations that will gain the most from selective immunotherapy, as current immunosuppression for these disorders leads to significant morbidity and mortality (81–84).

## Challenges for the Future

The rapid increase in our understanding of T-cell metabolism offers exciting opportunities and presents several challenges. The majority of initial metabolic studies were performed *in vitro* and many of these paradigms and results may not reflect *in vivo* biologic reality, which needs to be addressed. In addition, metabolic adaptation is by necessity a dynamic and responsive process, and conditions both inside and outside the T-cell change dramatically from one moment to the next. Thus, a thorough view of metabolism in any model needs to incorporate data and observations from multiple time points of analysis. Third, we know very little about the molecular machinery that drives adoption of metabolic phenotypes, particularly *in vivo*. Follow-up studies will need to not only identify proteins important in T-cell metabolism, but also define how the dependence on these factors changes during the course of an immune response. The field also needs metabolic activators and inhibitors with increased specificity, both for study purposes and the potential for therapeutic intervention (43). Finally, the study of T-cell metabolism needs to expand to better include the human immune system, particularly in the context of immune-mediated disorders. Future studies in human beings will benefit both from clinically based flux analysis using labeled metabolites such as ^13^C-glucose or ^13^C-palmitate (85, 86) and the great variety of pathways and compounds being discovered in the field of cancer therapy (79, 87–90).

## Conclusion

T-cell activation represents a time of significant energetic stress and cells must respond to this challenge by reprograming their metabolism to keep pace with increased metabolic demands. During a murine model of GVHD, effector T-cells increase their dependence on oxidative metabolism and FAO. Adoption of these pathways is likely due to environmental factors present at the time of T-cell recovery and analysis, including the ubiquity and duration of antigen exposure. Thus, T-cell differentiation status (e.g., memory versus effector) is not the sole arbitrator of metabolic phenotype, and our data suggest that effector T-cells will instead respond as necessary to meet the metabolic demands placed upon them, including upregulation of FAO. Future studies will determine how broadly findings on allogeneic T-cells can be applied to other models of chronic antigen exposure. Finally, these hypotheses must be tested in human immune responses, where a better understanding of T-cell metabolism might lead to enhanced vaccine strategies, improved anti-cancer responses, novel interventions against autoimmunity, and better post-transplant immunotherapy.

## Conflict of Interest Statement

The author declares that the research was conducted in the absence of any commercial or financial relationships that could be construed as a potential conflict of interest.

## References

[1] Zhang N Bevan MJ. CD8(+) T cells: foot soldiers of the immune system. Immunity (2011) 35:161–8. 10.1016/j.immuni.2011.07.010 21867926 PMC3303224

[2] Kaech SM Cui W. Transcriptional control of effector and memory CD8+ T cell differentiation. Nat Rev Immunol (2012) 12:749–61. 10.1038/nri3307 23080391 PMC4137483

[3] Badovinac VP Haring JS Harty JT. Initial T cell receptor transgenic cell precursor frequency dictates critical aspects of the CD8(+) T cell response to infection. Immunity (2007) 26:827–41. 10.1016/j.immuni.2007.04.013 17555991 PMC1989155

[4] Yoon H Kim TS Braciale TJ. The cell cycle time of CD8+ T cells responding in vivo is controlled by the type of antigenic stimulus. PLoS One (2010) 5:e15423. 10.1371/journal.pone.0015423 21079741 PMC2975678

[5] Fox CJ Hammerman PS Thompson CB. Fuel feeds function: energy metabolism and the T-cell response. Nat Rev Immunol (2005) 5:844–52. 10.1038/nri1710 16239903

[6] Jones RG Thompson CB. Revving the engine: signal transduction fuels T cell activation. Immunity (2007) 27:173–8. 10.1016/j.immuni.2007.07.008 17723208

[7] Pollizzi KN Powell JD. Integrating canonical and metabolic signalling programmes in the regulation of T cell responses. Nat Rev Immunol (2014) 14:435–46. 10.1038/nri3701 24962260 PMC4390057

[8] Pearce EL. Metabolism in T cell activation and differentiation. Curr Opin Immunol (2010) 22:314–20. 10.1016/j.coi.2010.01.018 20189791 PMC4486663

[9] Gerriets VA Rathmell JC. Metabolic pathways in T cell fate and function. Trends Immunol (2012) 33:168–73. 10.1016/j.it.2012.01.010 22342741 PMC3319512

[10] Pearce EL Poffenberger MC Chang CH Jones RG. Fueling immunity: insights into metabolism and lymphocyte function. Science (2013) 342:1242454. 10.1126/science.1242454 24115444 PMC4486656

[11] Maciver NJ Michalek RD Rathmell JC Metabolic regulation of T lymphocytes. Annu Rev Immunol (2013) 31:259–83 10.1146/annurev-immunol-032712-095956 23298210 PMC3606674

[12] Brand K. Glutamine and glucose metabolism during thymocyte proliferation. Pathways of glutamine and glutamate metabolism. Biochem J (1985) 228:353–61. 2861809 10.1042/bj2280353 PMC1144993

[13] Frauwirth KA Riley JL Harris MH Parry RV Rathmell JC Plas DR The CD28 signaling pathway regulates glucose metabolism. Immunity (2002) 16:769–77. 10.1016/S1074-7613(02)00323-0 12121659

[14] Maciver NJ Jacobs SR Wieman HL Wofford JA Coloff JL Rathmell JC. Glucose metabolism in lymphocytes is a regulated process with significant effects on immune cell function and survival. J Leukoc Biol (2008) 84:949–57. 10.1189/jlb.0108024 18577716 PMC2638731

[15] Macintyre AN Gerriets VA Nichols AG Michalek RD Rudolph MC Deoliveira D The glucose transporter Glut1 is selectively essential for CD4 T cell activation and effector function. Cell Metab (2014) 20:61–72. 10.1016/j.cmet.2014.05.004 24930970 PMC4079750

[16] Jacobs SR Herman CE Maciver NJ Wofford JA Wieman HL Hammen JJ Glucose uptake is limiting in T cell activation and requires CD28-mediated Akt-dependent and independent pathways. J Immunol (2008) 180:4476–86. 10.4049/jimmunol.180.7.4476 18354169 PMC2593791

[17] Michalek RD Gerriets VA Jacobs SR Macintyre AN Maciver NJ Mason EF Cutting edge: distinct glycolytic and lipid oxidative metabolic programs are essential for effector and regulatory CD4+ T cell subsets. J Immunol (2011) 186:3299–303. 10.4049/jimmunol.1003613 21317389 PMC3198034

[18] Yaqoob P Calder PC. Glutamine requirement of proliferating T lymphocytes. Nutrition (1997) 13:646–51. 10.1016/S0899-9007(97)83008-0 9263257

[19] Cummings JH Pomare EW Branch WJ Naylor CP Macfarlane GT. Short chain fatty acids in human large intestine, portal, hepatic and venous blood. Gut (1987) 28:1221–7. 10.1136/gut.28.10.1221 3678950 PMC1433442

[20] Nakaya M Xiao Y Zhou X Chang JH Chang M Cheng X Inflammatory T cell responses rely on amino acid transporter ASCT2 facilitation of glutamine uptake and mTORC1 kinase activation. Immunity (2014) 40:692–705. 10.1016/j.immuni.2014.04.007 24792914 PMC4074507

[21] Opitz CA Litzenburger UM Sahm F Ott M Tritschler I Trump S An endogenous tumour-promoting ligand of the human aryl hydrocarbon receptor. Nature (2011) 478:197–203. 10.1038/nature10491 21976023

[22] Sinclair LV Rolf J Emslie E Shi YB Taylor PM Cantrell DA. Control of amino-acid transport by antigen receptors coordinates the metabolic reprogramming essential for T cell differentiation. Nat Immunol (2013) 14:500–8. 10.1038/ni.2556 23525088 PMC3672957

[23] Nicklin P Bergman P Zhang B Triantafellow E Wang H Nyfeler B Bidirectional transport of amino acids regulates mTOR and autophagy. Cell (2009) 136:521–34. 10.1016/j.cell.2008.11.044 19203585 PMC3733119

[24] Munn DH Zhou M Attwood JT Bondarev I Conway SJ Marshall B Prevention of allogeneic fetal rejection by tryptophan catabolism. Science (1998) 281:1191–3. 10.1126/science.281.5380.1191 9712583

[25] Munn DH Shafizadeh E Attwood JT Bondarev I Pashine A Mellor AL. Inhibition of T cell proliferation by macrophage tryptophan catabolism. J Exp Med (1999) 189:1363–72. 10.1084/jem.189.9.1363 10224276 PMC2193062

[26] Mellor AL Munn DH IDO expression by dendritic cells: tolerance and tryptophan catabolism. Nat Rev Immunol (2004) 4:762–74 10.1038/nri1457 15459668

[27] Furusawa Y Obata Y Fukuda S Endo TA Nakato G Takahashi D Commensal microbe-derived butyrate induces the differentiation of colonic regulatory T cells. Nature (2013) 504:446–50. 10.1038/nature12721 24226770

[28] Smith PM Howitt MR Panikov N Michaud M Gallini CA Bohlooly YM The microbial metabolites, short-chain fatty acids, regulate colonic Treg cell homeostasis. Science (2013) 341:569–73. 10.1126/science.1241165 23828891 PMC3807819

[29] Eberl M Moser B. Monocytes and gammadelta T cells: close encounters in microbial infection. Trends Immunol (2009) 30:562–8. 10.1016/j.it.2009.09.001 19853512

[30] Kleinewietfeld M Manzel A Titze J Kvakan H Yosef N Linker RA Sodium chloride drives autoimmune disease by the induction of pathogenic TH17 cells. Nature (2013) 496:518–22. 10.1038/nature11868 23467095 PMC3746493

[31] Wu C Yosef N Thalhamer T Zhu C Xiao S Kishi Y Induction of pathogenic TH17 cells by inducible salt-sensing kinase SGK1. Nature (2013) 496:513–7. 10.1038/nature11984 23467085 PMC3637879

[32] Wulff P Vallon V Huang DY Volkl H Yu F Richter K Impaired renal Na(+) retention in the sgk1-knockout mouse. J Clin Invest (2002) 110:1263–8. 10.1172/JCI0215696 12417564 PMC151609

[33] Ardawi MS Newsholme EA. Metabolism of ketone bodies, oleate and glucose in lymphocytes of the rat. Biochem J (1984) 221:255–60. 6466315 10.1042/bj2210255 PMC1144027

[34] Frauwirth KA Thompson CB. Regulation of T lymphocyte metabolism. J Immunol (2004) 172:4661–5. 10.4049/jimmunol.172.8.4661 15067038

[35] Pearce EL Walsh MC Cejas PJ Harms GM Shen H Wang LS Enhancing CD8 T-cell memory by modulating fatty acid metabolism. Nature (2009) 460:103–7. 10.1038/nature08097 19494812 PMC2803086

[36] Hardie DG Carling D. The AMP-activated protein kinase – fuel gauge of the mammalian cell? Eur J Biochem (1997) 246:259–73. 10.1111/j.1432-1033.1997.00259.x 9208914

[37] Hardie DG. Energy sensing by the AMP-activated protein kinase and its effects on muscle metabolism. Proc Nutr Soc (2011) 70:92–9. 10.1017/S0029665110003915 21067629

[38] O’Neill HM Holloway GP Steinberg GR. AMPK regulation of fatty acid metabolism and mitochondrial biogenesis: implications for obesity. Mol Cell Endocrinol (2013) 366:135–51. 10.1016/j.mce.2012.06.019 22750049

[39] van der Windt GJ Everts B Chang CH Curtis JD Freitas TC Amiel E Mitochondrial respiratory capacity is a critical regulator of CD8+ T cell memory development. Immunity (2012) 36:68–78. 10.1016/j.immuni.2011.12.007 22206904 PMC3269311

[40] Rolf J Zarrouk M Finlay DK Foretz M Viollet B Cantrell DA. AMPKalpha1: a glucose sensor that controls CD8 T-cell memory. Eur J Immunol (2013) 43:889–96. 10.1002/eji.201243008 23310952 PMC3734624

[41] Wong AK Howie J Petrie JR Lang CC. AMP-activated protein kinase pathway: a potential therapeutic target in cardiometabolic disease. Clin Sci (Lond) (2009) 116:607–20. 10.1042/CS20080066 19275766 PMC2762688

[42] Buler M Aatsinki SM Izzi V Uusimaa J Hakkola J. SIRT5 is under the control of PGC-1alpha and AMPK and is involved in regulation of mitochondrial energy metabolism. FASEB J (2014) 28:3225–37. 10.1096/fj.13-245241 24687991

[43] Vincent EE Coelho PP Blagih J Griss T Viollet B Jones RG. Differential effects of AMPK agonists on cell growth and metabolism. Oncogene (2014). 10.1038/onc.2014.301 25241895 PMC4980123

[44] Owen MR Doran E Halestrap AP. Evidence that metformin exerts its anti-diabetic effects through inhibition of complex 1 of the mitochondrial respiratory chain. Biochem J (2000) 348(Pt 3):607–14. 10.1042/0264-6021:3480607 10839993 PMC1221104

[45] Ben Sahra I Le Marchand-Brustel Y Tanti JF Bost F. Metformin in cancer therapy: a new perspective for an old antidiabetic drug? Mol Cancer Ther (2010) 9:1092–9. 10.1158/1535-7163.MCT-09-1186 20442309

[46] Sukumar M Liu J Ji Y Subramanian M Crompton JG Yu Z Inhibiting glycolytic metabolism enhances CD8+ T cell memory and antitumor function. J Clin Invest (2013) 123:4479–88. 10.1172/JCI69589 24091329 PMC3784544

[47] Gubser PM Bantug GR Razik L Fischer M Dimeloe S Hoenger G Rapid effector function of memory CD8+ T cells requires an immediate-early glycolytic switch. Nat Immunol (2013) 14:1064–72. 10.1038/ni.2687 23955661

[48] Nakada D Saunders TL Morrison SJ. Lkb1 regulates cell cycle and energy metabolism in haematopoietic stem cells. Nature (2010) 468:653–8. 10.1038/nature09571 21124450 PMC3059717

[49] Wahl DR Byersdorfer CA Ferrara JL Opipari AW Jr. Glick GD. Distinct metabolic programs in activated T cells: opportunities for selective immunomodulation. Immunol Rev (2012) 249:104–15. 10.1111/j.1600-065X.2012.01148.x 22889218 PMC3422770

[50] McNamee EN Korns Johnson D Homann D Clambey ET. Hypoxia and hypoxia-inducible factors as regulators of T cell development, differentiation, and function. Immunol Res (2013) 55:58–70. 10.1007/s12026-012-8349-8 22961658 PMC3919451

[51] Lum JJ Bui T Gruber M Gordan JD Deberardinis RJ Covello KL The transcription factor HIF-1alpha plays a critical role in the growth factor-dependent regulation of both aerobic and anaerobic glycolysis. Genes Dev (2007) 21:1037–49. 10.1101/gad.1529107 17437992 PMC1855230

[52] Dziurla R Gaber T Fangradt M Hahne M Tripmacher R Kolar P Effects of hypoxia and/or lack of glucose on cellular energy metabolism and cytokine production in stimulated human CD4+ T lymphocytes. Immunol Lett (2010) 131:97–105. 10.1016/j.imlet.2010.02.008 20206208

[53] Atkuri KR Herzenberg LA Niemi AK Cowan T Herzenberg LA. Importance of culturing primary lymphocytes at physiological oxygen levels. Proc Natl Acad Sci U S A (2007) 104:4547–52. 10.1073/pnas.0611732104 17360561 PMC1838638

[54] Tripmacher R Gaber T Dziurla R Haupl T Erekul K Grutzkau A Human CD4(+) T cells maintain specific functions even under conditions of extremely restricted ATP production. Eur J Immunol (2008) 38:1631–42. 10.1002/eji.200738047 18493983

[55] Glick GD Rossignol R Lyssiotis CA Wahl D Lesch C Sanchez B Anaplerotic metabolism of alloreactive T cells provides a metabolic approach to treat graft-versus-host disease. J Pharmacol Exp Ther (2014) 351:298–307. 10.1124/jpet.114.218099 25125579 PMC4201277

[56] Shlomchik WD. Graft-versus-host disease. Nat Rev Immunol (2007) 7:340–52. 10.1038/nri2000 17438575

[57] Ferrara JL Levine JE Reddy P Holler E. Graft-versus-host disease. Lancet (2009) 373:1550–61. 10.1016/S0140-6736(09)60237-3 19282026 PMC2735047

[58] Paczesny S Hanauer D Sun Y Reddy P. New perspectives on the biology of acute GVHD. Bone Marrow Transplant (2010) 45:1–11. 10.1038/bmt.2009.328 19946340 PMC7793552

[59] Gatza E Wahl DR Opipari AW Sundberg TB Reddy P Liu C Manipulating the bioenergetics of alloreactive T cells causes their selective apoptosis and arrests graft-versus-host disease. Sci Transl Med (2011) 3:67ra68. 10.1126/scitranslmed.3001975 21270339 PMC3364290

[60] Kuhnke A Burmester GR Krauss S Buttgereit F. Bioenergetics of immune cells to assess rheumatic disease activity and efficacy of glucocorticoid treatment. Ann Rheum Dis (2003) 62:133–9. 10.1136/ard.62.2.133 12525382 PMC1754434

[61] Nagy G Barcza M Gonchoroff N Phillips PE Perl A. Nitric oxide-dependent mitochondrial biogenesis generates Ca2+ signaling profile of lupus T cells. J Immunol (2004) 173:3676–83. 10.4049/jimmunol.173.6.3676 15356113 PMC4034140

[62] Reuter SE Evans AM. Carnitine and acylcarnitines: pharmacokinetic, pharmacological and clinical aspects. Clin Pharmacokinet (2012) 51:553–72. 10.2165/11633940-000000000-00000 22804748

[63] Byersdorfer CA Tkachev V Opipari AW Goodell S Swanson J Sandquist S Effector T cells require fatty acid metabolism during murine graft-versus-host disease. Blood (2013) 122:3230–7. 10.1182/blood-2013-04-495515 24046012 PMC3814737

[64] Shriver LP Manchester M. Inhibition of fatty acid metabolism ameliorates disease activity in an animal model of multiple sclerosis. Sci Rep (2011) 1:79. 10.1038/srep00079 22355598 PMC3216566

[65] Balaban RS Nemoto S Finkel T. Mitochondria, oxidants, and aging. Cell (2005) 120:483–95. 10.1016/j.cell.2005.02.001 15734681

[66] Sariban-Sohraby S Magrath IT Balaban RS. Comparison of energy metabolism in human normal and neoplastic (Burkitt’s lymphoma) lymphoid cells. Cancer Res (1983) 43:4662–4. 6883323

[67] Shi LZ Wang R Huang G Vogel P Neale G Green DR HIF1alpha-dependent glycolytic pathway orchestrates a metabolic checkpoint for the differentiation of TH17 and Treg cells. J Exp Med (2011) 208:1367–76. 10.1084/jem.20110278 21708926 PMC3135370

[68] Wahl DR Petersen B Warner R Richardson BC Glick GD Opipari AW. Characterization of the metabolic phenotype of chronically activated lymphocytes. Lupus (2010) 19:1492–501. 10.1177/0961203310373109 20647250

[69] Sena LA Li S Jairaman A Prakriya M Ezponda T Hildeman DA Mitochondria are required for antigen-specific T cell activation through reactive oxygen species signaling. Immunity (2013) 38:225–36. 10.1016/j.immuni.2012.10.020 23415911 PMC3582741

[70] Parry RV Chemnitz JM Frauwirth KA Lanfranco AR Braunstein I Kobayashi SV CTLA-4 and PD-1 receptors inhibit T-cell activation by distinct mechanisms. Mol Cell Biol (2005) 25:9543–53. 10.1128/MCB.25.21.9543-9553.2005 16227604 PMC1265804

[71] Kudo N Barr AJ Barr RL Desai S Lopaschuk GD. High rates of fatty acid oxidation during reperfusion of ischemic hearts are associated with a decrease in malonyl-CoA levels due to an increase in 5’-AMP-activated protein kinase inhibition of acetyl-CoA carboxylase. J Biol Chem (1995) 270:17513–20. 10.1074/jbc.270.29.17513 7615556

[72] Mihaylova MM Shaw RJ. The AMPK signalling pathway coordinates cell growth, autophagy and metabolism. Nat Cell Biol (2011) 13:1016–23. 10.1038/ncb2329 21892142 PMC3249400

[73] Maciver NJ Blagih J Saucillo DC Tonelli L Griss T Rathmell JC The liver kinase B1 is a central regulator of T cell development, activation, and metabolism. J Immunol (2011) 187:4187–98. 10.4049/jimmunol.1100367 21930968 PMC3206094

[74] Lee J Walsh MC Hoehn KL James DE Wherry EJ Choi Y. Regulator of fatty acid metabolism, acetyl coenzyme a carboxylase 1, controls T cell immunity. J Immunol (2014) 192:3190–9. 10.4049/jimmunol.1302985 24567531 PMC3965631

[75] Patra KC Hay N The pentose phosphate pathway and cancer. Trends Biochem Sci (2014) 39:347–54 10.1016/j.tibs.2014.06.005 25037503 PMC4329227

[76] Berod L Friedrich C Nandan A Freitag J Hagemann S Harmrolfs K De novo fatty acid synthesis controls the fate between regulatory T and T helper 17 cells. Nat Med (2014) 20:1327–33. 10.1038/nm.3704 25282359

[77] Quintana FJ Basso AS Iglesias AH Korn T Farez MF Bettelli E Control of T(reg) and T(H)17 cell differentiation by the aryl hydrocarbon receptor. Nature (2008) 453:65–71. 10.1038/nature06880 18362915

[78] Dang EV Barbi J Yang HY Jinasena D Yu H Zheng Y Control of T(H)17/T(reg) balance by hypoxia-inducible factor 1. Cell (2011) 146:772–84. 10.1016/j.cell.2011.07.033 21871655 PMC3387678

[79] Possemato R Marks KM Shaul YD Pacold ME Kim D Birsoy K Functional genomics reveal that the serine synthesis pathway is essential in breast cancer. Nature (2011) 476:346–50. 10.1038/nature10350 21760589 PMC3353325

[80] Kidani Y Elsaesser H Hock MB Vergnes L Williams KJ Argus JP Sterol regulatory element-binding proteins are essential for the metabolic programming of effector T cells and adaptive immunity. Nat Immunol (2013) 14:489–99. 10.1038/ni.2570 23563690 PMC3652626

[81] Baddley JW Stroud TP Salzman D Pappas PG. Invasive mold infections in allogeneic bone marrow transplant recipients. Clin Infect Dis (2001) 32:1319–24. 10.1086/319985 11303267

[82] Leather HL Wingard JR. Infections following hematopoietic stem cell transplantation. Infect Dis Clin North Am (2001) 15:483–520. 10.1016/S0891-5520(05)70157-4 11447707

[83] Doligalski CT Benedict K Cleveland AA Park B Derado G Pappas PG Epidemiology of invasive mold infections in lung transplant recipients. Am J Transplant (2014) 14:1328–33. 10.1111/ajt.12691 24726020 PMC4158712

[84] Grubbs JA Baddley JW. Pneumocystis jirovecii pneumonia in patients receiving tumor-necrosis-factor-inhibitor therapy: implications for chemoprophylaxis. Curr Rheumatol Rep (2014) 16:445. 10.1007/s11926-014-0445-4 25182673

[85] Egli L Lecoultre V Theytaz F Campos V Hodson L Schneiter P Exercise prevents fructose-induced hypertriglyceridemia in healthy young subjects. Diabetes (2013) 62:2259–65. 10.2337/db12-1651 23674606 PMC3712038

[86] Koutsari C Mundi MS Ali AH Patterson BW Jensen MD. Systemic free fatty acid disposal into very low-density lipoprotein triglycerides. Diabetes (2013) 62:2386–95. 10.2337/db12-1557 23434937 PMC3712051

[87] Bonnet S Archer SL Allalunis-Turner J Haromy A Beaulieu C Thompson R A mitochondria-K+ channel axis is suppressed in cancer and its normalization promotes apoptosis and inhibits cancer growth. Cancer Cell (2007) 11:37–51. 10.1016/j.ccr.2006.10.020 17222789

[88] Michelakis ED Sutendra G Dromparis P Webster L Haromy A Niven E Metabolic modulation of glioblastoma with dichloroacetate. Sci Transl Med (2010) 2:31ra34. 10.1126/scitranslmed.3000677 20463368

[89] Schulze A Harris AL. How cancer metabolism is tuned for proliferation and vulnerable to disruption. Nature (2012) 491:364–73. 10.1038/nature11706 23151579

[90] Galluzzi L Kepp O Vander Heiden MG Kroemer G Metabolic targets for cancer therapy. Nat Rev Drug Discov (2013) 12:829–46 10.1038/nrd4191 24113830

